# O Instituto de Investigação Científica de Angola: do Estado Novo à independência, 1955-1975

**DOI:** 10.1590/S0104-59702026000100006

**Published:** 2026-03-30

**Authors:** Soraia Santos Ferreira, Daniela de Matos

**Affiliations:** i Doutoranda em Estudos do Património, Faculdade de Letras/Universidade do Porto; CIBIO-BIOPOLIS/Universidade do Porto. Porto – Portugal. soraiafmsantos@gmail.com; ii Investigadora júnior, Centro de Arqueologia/Universidade de Lisboa; Museu de História Natural e da Ciência/Universidade de Lisboa. Lisboa – Portugal. dmatos@campus.ul.pt

**Keywords:** História da ciência, Instituições, Colonialismo, Portugal, Angola, History of science, Institutions, Colonialism, Portugal, Angola

## Abstract

O Instituto de Investigação Científica de Angola foi uma instituição colonial portuguesa criada em 1955. Como instrumento de ocupação portuguesa em África durante o Estado Novo, o instituto desempenhou um papel determinante na promoção da ciência e de atividades de investigação nas ciências naturais e humanas, dando origem a organismos que persistem na atualidade como são os museus nacionais e regionais de Angola. Neste artigo, analisamos documentação inédita para compreender a história do instituto desde a sua fundação até ao seu declínio e transformação.

## Abstract (en)

The Instituto de Investigação Científica de Angola [Angola Institute of Scientific Research] was a Portuguese colonial institution created in 1955. As an instrument of Portugal’s occupation in Africa during the New State period, it played a determining role in promoting science and research activities in the natural sciences and humanities, giving rise to organizations that still exist today, such as Angola’s regional and national museums. In this article we analyze new documentation to understand the history of the institute, from its founding to its decline and transformation.

O Instituto de Investigação Científica de Angola (IICA) entrou em funcionamento em 1957 e desenvolveu atividade até à independência de Angola, em 1975. Como instituição desenhada e gerida pelo Ministério do Ultramar português, o IICA destacou-se na investigação científica sobre a sociedade e o território angolano durante o período colonial do Estado Novo em Portugal (1936-1974).

O instituto deu origem a organismos que persistem na atualidade, como a maioria dos museus nacionais e regionais cujos acervos resultam da herança das organizações e instituições coloniais. O presente trabalho decorre de pesquisa em arquivos portugueses e angolanos e tem como base fontes publicadas e primárias. Em Portugal, foram consultadas as bibliotecas das Faculdades de Letras e dos Museus de História Natural das Universidades de Lisboa, Porto e Coimbra. Foi tratada arquivisticamente para este trabalho a correspondência trocada entre a Junta de Investigações do Ultramar e o IICA, herdada dos arquivos do Instituto de Investigação Científica Tropical e guardada agora no Arquivo dos Serviços Centrais da Universidade de Lisboa. Foram também consultados o Arquivo do Instituto de Alta Cultura e o Arquivo Pessoal de Fernando Mouta no Camões I.P., em Lisboa.

Em Angola, a consulta foi realizada na Biblioteca Nacional e no Arquivo Nacional, no Arquivo Lúcio Lara-Tchiweka, no Arquivo Provincial da Huíla, na Biblioteca e no Depósito do Museu Regional da Huíla, na Biblioteca do Instituto Superior de Ciências da Educação da Huíla (ISCED), no Arquivo da Imprensa Nacional e no Centro Nacional de Investigação Científica (CNIC). No entanto, a informação disponível em Luanda revelou-se limitada. No caso da Biblioteca Nacional e do Arquivo Nacional, assim como da Imprensa Nacional, a informação disponível consiste essencialmente em monografias e boletins editados pelo IICA. No CNIC, onde funcionava o IICA, o arquivo está inacessível e não existe um inventário da documentação. No Arquivo e na Biblioteca Provincial da Huíla, no Lubango, onde funcionava o antigo centro do IICA de Sá da Bandeira, encontrou-se a correspondência com o primeiro conservador do museu, Alberto Machado Cruz. No ISCED consultou-se a documentação do Arquivo de Botânica e Zoologia, que inclui cadernos de campo do IICA.

Com a informação por ora disponível foi analisada a atividade do IICA na génese de instituições de ciência e património em Angola, para responder à questão de qual foi o seu papel no desenvolvimento de estruturas laboratoriais, museus e coleções públicas. Neste artigo, debruçamo-nos sobre as origens do IICA, os antecedentes da sua oficialização em 1955, a sua organização e produção científica, reunindo informação dispersa que permita compreender a história de uma instituição, embora efémera, com uma duração não superior a 20 anos, no quadro da administração colonial portuguesa, mas que empreendeu projetos de pesquisa e formou espólios cujo destino se pretende aqui reconstituir.

## Os antecedentes da fundação do IICA

O IICA foi oficializado a 7 de março de 1955, com o decreto fundacional dos Institutos de Investigação Científica e Medicina Tropical em Angola e Moçambique (Portaria n.40078, 7 mar. 1955). Em nove artigos definiu-se missão, gestão e orgânica administrativa dos institutos, enfatizando a direta dependência do Ministério do Ultramar por meio dos governos-gerais em Luanda e Maputo, assim como dos pareceres da Junta de Investigações do Ultramar em Lisboa. A missão era clara e definida no primeiro artigo: “Fomentar a cultura e promover e auxiliar os trabalhos de investigação científica naqueles territórios especialmente nos domínios da geografia, história natural e das ciências humanas” (Portaria n.40078, 7 mar. 1955). O decreto de fundação materializa a “Ocupação científica do Ultramar”, que vinha a ser promovida e debatida pela elite política e académica portuguesa desde os anos 1930. Luiz Wittnich Carriso (1934, p.16), em conferência na primeira Exposição Colonial Portuguesa no Porto, afirmava que

a colonização tem de ser feita cientificamente, dando a esta palavra o seu significado mais vasto ... a criação de um instituto de investigação científica colonial ... Com o decorrer do tempo, e o progressivo desenvolvimento destes serviços, alargar-se-iam os quadros dos institutos interessados, criando-se, assim, um importante corpo de investigadores ... . A organização do nosso museu colonial seria o coroamento deste plano. Não temos hoje, nem material, nem pessoal para esse empreendimento: mas dentro de alguns anos, estou certo de que isso seria possível, desde que lançássemos já mãos à obra.

Similarmente, Fernando Mouta (1936, p.3), engenheiro da Seção de Indústria e Minas da colónia de Angola, antigo membro da Missão Geológica e presidente da Câmara Municipal de Luanda entre 1932 e 1939, pronunciou-se sobre a criação de um Instituto de Investigação Científica Colonial, considerando

indispensável e urgente que o Estado Novo no seu programa de ressurgimento nacional promova a ocupação científica debaixo do Império colonial pois muito pouco tinha sido feito pelos governos da metrópole para resolver este fundamental problema, … e a obra dos estrangeiros se começava a sobrepor pois somente duas tentativas tinham sido realizadas até então de explorar os territórios coloniais.

Referia-se à primeira missão de botânica de Angola (1853-1861) pelo austríaco Friedrich Welwitsch, em nome do reino de Portugal, e às missões de reconhecimento geológico em Cabo Verde, Moçambique e Angola. Excluía, porém, as missões da Escola de Medicina Tropical a Angola, São Tomé, Príncipe, Guiné e Moçambique (Amaral, 2012; Havik, 2014; Varanda, 2014), ou a missão hidrográfica de Moçambique (1929) ou a missão antropológica financiada pela Junta de Educação Nacional (1929-1936) e postumamente pelo Instituto de Alta Cultura, com uma missão a Angola em 1934 por António de Almeida, e uma missão a Moçambique por Joaquim Santos Júnior, em 1936 (Martins, 2013, 2020).

Essas reivindicações sugeriam a descentralização e diversificação dos projetos até então concentrados na Comissão de Cartografia, sucedida pela Junta das Missões Geográficas e das Investigações Coloniais (Decreto-lei n.26180, 7 jan. 1936). O novo organismo assumiu a tutela e coordenação das missões científicas às colónias, projetando o domínio da população por meio do conhecimento da sua história, sociedade e política (Martins, Albino, 2010). Com a Junta nasceu um novo impulso que se traduziu na criação de novas missões científicas às colónias e no relançamento de outras já existentes (Castelo, 2012; Costa, 2013). Passou a contemplar, no seu espectro, novas temáticas das ciências sociais e humanas, especialmente no domínio da antropologia e etnografia. Almejava-se resgatar Portugal do atraso em relação a outras potências imperiais no “controlo científico” dos territórios e populações colonizadas.

No caso específico de Angola, as deficiências na estrutura de conservação do património natural e cultural eram notórias para a administração colonial *in loco*. Em 1936, Mouta (1936) defendeu a criação de um Museu de História Natural em Angola, apontando a igual necessidade de promulgar uma legislação que proibisse a saída de coleções de objetos indígenas das colónias. No fundo, era uma nota de crítica ao poder central, sucedendo à notícia de que o ministro dos Negócios Estrangeiros de Portugal tinha assistido recentemente à inauguração das salas de Angola no Museu de História Natural de La Chaux-de Fonds, na Suíça, onde se expunham vinte mil exemplares faunísticos, três mil objetos gentílicos e mais de 2.500 fotografias recolhidos pelo curador e conservador do museu Albert Monard, que rivalizava com o acervo do Museu Bocage em Lisboa (Mouta, 1936). Assim, os estudos movidos por si próprio em Angola (Mouta, O’Donnell, 1933) ou por entidades privadas, como foi o caso dos projetos científicos da Companhia dos Diamantes de Angola, por intermédio do Museu do Dundo (Figueiredo, 2020), constituíam pequenos investimentos.

A junta viria colmatar, de alguma forma, essas lacunas tanto embora os planos se projetassem modestos, como ficou claro no Plano de Investigação Científica Colonial apresentado pela presidência da junta ao ministro das Colónias em 1941. Nesse documento, Bacellar Bebiano pronunciava-se sobre o papel da junta no prestígio nacional em que “a Junta … entende que, sem maior demora, se devem intensificar, ainda que de forma modesta, os trabalhos de investigação científica nas colónias” (Agência Geral das Colónias, 1945, p.13).

De acordo com o plano, a tutela das missões era do governo português, sendo apoiadas pelas administrações coloniais e geridas pelos académicos que tinham a autorização concedida por parte das entidades governamentais (Costa, 2013). Além da necessidade do reconhecimento dos territórios e das populações locais, a intenção de contribuir para a melhoria do quotidiano das comunidades sobressaía na retórica política e intelectual da época, que se pretendia mais aproximada do contexto internacional. Iniciativas como o Institut Fondamental de Afrique Noire (1936) em Dakar, no Senegal, na esfera do império francês, e o Rhodes-Livingstone Institute (1937) em Lusaka, na Zâmbia (Schumaker, 2001), exclusivamente dedicado à antropologia. Em Portugal, os estudos antropológicos adquiriram um papel de destaque no domínio das ciências humanas, procurando caracterizar biologicamente e mentalmente os indígenas, desde as suas fisiologias até aos seus hábitos e aspirações (Poloni, Moraes, 2015), com o objetivo de controlar cientificamente a população e reforçar o poder colonial (Roque, Marques, 2011). É nesse quadro que se insere a ascensão política de António Mendes Corrêa no Ministério do Ultramar, primeiro a encabeçar a investigação antropológica no Ultramar a partir do Instituto de Antropobiologia da Faculdade de Ciências do Porto, com os seus discípulos António de Almeida e Joaquim Santos Júnior. Depois, participou na origem da Junta das Missões Geográficas e Investigações Coloniais (JMGIC) e do Instituto de Alta Cultura, assim como na reforma do sistema educativo direcionado para os “assuntos das colónias” (Matos, 2012).

As maiores preocupações recaíam em questões financeiras para a execução do plano, relativas ao investimento necessário para tal, um desafio partilhado por outras potências coloniais europeias (Agência Geral das Colónias, 1945, p.99). A falta de investimento afetava áreas de relevância nuclear para a manutenção do poder colonial, como o mapeamento do território. Sobre esse assunto, o general Norton de Matos (29 mar. 1944, p.6), administrador emérito das posses coloniais e responsável pela criação dos Serviços Geográficos de Angola, apontava a necessidade de concluir a cartografia de Angola “sobretudo no território entre o deserto do Namibe e o planalto, onde as condições de extrema aridez limitavam o reconhecimento, e, por conseguinte, a sua valorização económica” (Matos, 29 mar. 1944, p.6), pois, apesar das numerosas ações pontuais para o reconhecimento dos territórios colonizados já em marcha, apenas campanhas pouco sistemáticas tinham sido desenvolvidas.

A partir de 1945, a atividade da JMGIC assume um novo fulgor em face da pressão internacional no desfecho da Segunda Guerra Mundial e com a recusa no ingresso de Portugal à Organização das Nações Unidas devido ao colonialismo e ao surgimento de movimentos de libertação e independência (Benot, 1981). O Estado Novo procurou aproximar-se das suas congéneres em nível diplomático, com a participação de Portugal no Conselho Científico Africano/Comissão de Cooperação Técnica ao Sul do Saara (CCA/CCTA) (Vigier, 1954). Procurava igualmente aproximar-se dos avanços científicos internacionais, modernizar e adaptar ao novo ambiente geopolítico (Ágoas, Castelo, 2019). Mendes Corrêa foi membro do CCA na qualidade de diretor da junta, cargo que assumiu em 1946, e acumulou a direção da Escola Superior Colonial, futuro Instituto Superior de Estudos Ultramarinos. Dois outros indivíduos representaram Portugal no CCA: Francisco Cabournac, do Instituto de Medicina Tropical, e Fernando Mouta. Este último representava Portugal nas comissões dedicadas à geologia, à estratigrafia e aos solos em representação dos Serviços de Geologia e Minas de Angola e Moçambique, que tinha ajudado a estabelecer.

Nos anos imediatamente anteriores ao decreto de fundação do IICA, a elite científica portuguesa esteve envolvida nas várias conferências e reuniões do CCA/CCTA. A realidade do atraso estrutural e académico em relação às outras potências europeias incomodou os membros-correspondentes, que regressaram com petições para produzir pesquisa de qualidade semelhante (Castelo, 2022). Nesse contexto, o IICA reforçava ocupação científica estratégica em África, e também a posição portuguesa na continuidade do colonialismo, ainda que adaptado ou transformado para suprimir a resistência (Alexandre, 2017).

A discussão do regulamento dos institutos dentro da junta começou em 1950, com duas versões anotadas e revistas pelo secretariado da presidência (Dossier, 1955-1973). A atribuição de verbas foi instruída por Mendes Corrêa em 1954 (Informação n.199/54, 6 out. 1954) e iniciava o processo de implementação dos institutos, decretados a 7 de março de 1955. Meses mais tarde, a nomeação dos diretores dos institutos oficializou o início das atividades (Informação n.572/56, 5 jan. 1956). Para Angola, foi nomeado Virgílio Cannas Martins, engenheiro da Estação Agronómica Nacional do Ministério da Economia. Nos primeiros meses dedicou-se à formação e à preparação para o cargo, tal como o seu congénere em Moçambique, José Emílio Pinto Lopes (Informação n.128/572/56, 2 maio 1956). Durante três meses, deslocou-se à Bélgica, Grã-Bretanha e França para fazer contatos e visitas técnicas.

## O funcionamento do IICA

Já em Luanda, o diretor do IICA se beneficiou do “estímulo e [das] facilidades” concedidos pelo Governo-geral de Angola, “especialmente de S. Ex. o governador-geral, que ao Instituto e aos seus problemas tem dispensado a melhor atenção e o maior auxílio, que se ficou devendo o impulso que permitiu que os seus serviços se encontrem em grande parte em atividade e outros, prontos para ação imediata” (Martins, 1958b). Inicialmente, a atividade do IICA recaiu sobretudo no recrutamento e preparação de funcionários, estabelecimento de relações e colaboração com outros organismos de investigação, levantamento de problemas locais, inventariação bibliográfica, apetrechamento de laboratórios e dos diversos serviços internos. O IICA pretendia oferecer “melhores e mais eficientes condições de trabalho, para se iniciar a contribuição do Instituto para o reconhecimento geral da província nos seus diversos aspetos ligados ao Homem, à Flora, à Fauna e ao Solo” (Martins, 1958a, p.14). Vários projetos de cooperação internacional estavam já a decorrer, como a elaboração do *Compectus Florae Angolensis* com o Museu Britânico, as pesquisas sobre o cafeeiro com os EUA, e a implementação do Secretariado Geral da Association pour l’Étude Taxonomique de la Flore d’Afrique Tropicale (Martins, 1958b). Em Portugal, as parcerias com “a quase totalidade dos organismos metropolitanos que se interessam pelos estudos dos problemas de Angola” (Martins, 1958a, p.15) incluíam os centros e institutos da Junta e as universidades. Cannas Martins também desempenhava funções no Centro de Estudos de Antropobiologia da Junta de Investigações do Ultramar (Decreto-lei n.44530, 21 ago. 1962), o que facilitaria essa ligação e o recrutamento de pessoal.

Inicialmente, o IICA foi organizado em divisões dedicadas à pesquisa sobre o meio ambiente. No primeiro relatório do diretor do IICA, a estrutura incluía uma Divisão de Estudos Florestais, de Botânica, de Entomologia e de Ornitologia (Martins, 1958a). As bolsas de estágio atribuídas e as campanhas realizadas nos primeiros anos dedicaram-se a medicina veterinária, estudos florestais e zoologia. Rapidamente seriam introduzidas outras divisões e a diversificação temática. Em 1959, o IICA tinha um Centro de Documentação Científica que albergava toda a bibliografia adquirida e produzida pelo instituto; uma Secção de Pedologia, dedicada à correção dos solos; a Secção de Ciências Biológicas; uma Secção de Botânica, e uma Divisão de Estudos Agronómicos. As ações prioritárias incluíam os estudos sobre citrinos, abacaxis, café, forragens, sisal e sacarina. Os Serviços de Economia Agrícola, dirigidos por Cruz Carvalho, dedicavam-se aos “processos de cultura entre os indígenas [e] os europeus”, e a Divisão dos Estudos Florestais, onde se estudava “a regeneração das florestas e a viabilidade da exploração” tendo já sido produzida celulose a partir da madeira de embondeiro (Agência Geral do Ultramar, 1959, p.295).

O IICA projetou também núcleos de pesquisa especializada fora de Luanda. Incluem-se os núcleos na Cela e Malanje, para pesquisas agronómicas e sociológicas, ou no Huambo em colaboração com a Junta dos Cereais (Agência Geral do Ultramar, 1959). Já o centro do IICA, em Sá da Bandeira (atual Lubango), já se encontrava a funcionar em 1961 e destinava-se a promover atividades na área da agronomia, botânica e zoologia, assim como apoiar missões sobre a antropologia, etnologia, arte e memória local, em grande medida lideradas ou apoiadas pelo primeiro conservador do Museu da Huíla, Alberto Damião Machado da Cruz, professor do Liceu Diogo Cão (atual Universidade Mandume Ya Ndemufayo).

O Museu da Huíla foi fundado em 1957 por Peixoto Correia com o objetivo de recolher, albergar, estudar e divulgar achados locais da paleontologia, arqueologia e etnografia. O acervo contém objetos recolhidos entre as comunidades do sul de Angola, assim como decorações e mobiliário oferecidos pela comunidade madeirense ou de tradição portuguesa. Machado da Cruz (1958, 1967, 1971) desenvolvia também uma “vila museológica” em Cangalongue, no Jau, Huíla, sobre a qual pouco se conhece para além da sua existência. Já à época, o Museu da Huíla integrava um vasto repertório sobre as comunidades locais, mas também sobre as atuais províncias do Namibe, Cunene e Cuando-Cubango. Era uma pesquisa independente das missões religiosas, que até então concentravam atividades nesse domínio (Agência Geral das Colónias, 1945, p.13). A delegação do IICA na Huíla corporizava a estratégia de descentralização necessária à ocupação efetiva dos territórios mais inóspitos, mas implicava a fusão e remodelação dos museus já existentes, como o Museu do Kongo, o Museu de Angola e o Museu da Huíla.

Após a integração no IICA, o Museu de Angola, na Fortaleza de S. Miguel de Luanda desde 8 de setembro de 1938, foi transferido para novas instalações em 1958, onde atualmente se localiza o Museu Nacional de História Natural. O museu foi construído de raiz para albergar e expor coleções desse cariz com coleções de moluscos, borboletas e conchas, algumas usadas como moeda na costa ocidental de África antes da chegada dos europeus. O ambiente expositivo do museu tentou recriar o *habitat* das espécies representadas, enquadrando elementos recolhidos pelos membros do IICA.

Na fase inicial do funcionamento do instituto, os corpos integrantes provinham de forma direta ou indireta dos quadros da Junta de Investigações do Ultramar ou teriam sido propostos por altos cargos da junta. A correspondência sugere que o recrutamento também se realizou por candidatura espontânea dos interessados, os quais foram aceites, na sua maioria. No avanço dos trabalhos científicos nas regiões do Sul participaram missões vindas diretamente da metrópole, desenvolvidas por membros da junta com o apoio obrigatório do IICA, que se debatia com a falta de recursos para assumir a logística de todos os trabalhos (Martins, 1968). Ainda assim acolheu e apoiou várias expedições, inclusive de estrangeiros (Urquhart, 1963). Mas nem todos os pedidos de pesquisa foram aprovados, como, por exemplo, a proposta de colaboração de John Desmond Clark, do Rhodes Livingstone Institute, à época em serviço no Museu do Dundo. Sua proposta foi chumbada pela direção da junta, de forma a proteger a imagem do Estado sobre a sua relação com os grupos locais “menos aculturados”, embora apoiada pelo parecer do diretor do Centro de Estudos de Antropobiologia (Almeida, 13 maio 1964), que imediatamente propõe a realização de uma campanha de estudos arqueológicos para o sudoeste de Angola (26 jul. 1964), que se viria a realizar somente em 1966, por Miguel Ramos (1970) em colaboração com membros do IICA.

Para a administração colonial, o sucesso da ciência colonial não podia depender das colaborações externas, pelo que urgia criar condições para a qualificação de quadros em Angola (Informação n.198/72/61, 6 set. 1961). Em carta à Comissão Executiva da Junta de Investigações do Ultramar, o diretor do IICA oferecia um balanço interno da atividade do instituto, considerando o plano de ocupação colonial e os princípios que presidiram a sua fundação, mas enfatizando a ausência de recursos financeiros e humanos para essa empreitada. Cannas Martins (8 jul. 1961, p.2) indicava que

foi possível: 1) Criar e manter algumas bases de estudo e investigação; 2) Iniciar o reconhecimento dos recursos naturais e valores da Província, nos diferentes aspectos ligados ao Homem e ao Meio; 3) Apoiar os serviços públicos e entidades particulares no estudo dos seus problemas mais especializados; 4) Fomentar a cultura na Província; e 5) Desenvolver as relações culturais e científicas com a metrópole e com o estrangeiro. … Porém as dificuldades de ordem material com que o Instituto lutou nos últimos tempos e, sobretudo os graves problemas ligados ao recrutamento e à estabilidade do pessoal não permitiram ainda a criação de núcleos permanentes de trabalhadores que garantam a continuidade na realização das tarefas que se pretendiam levar a cabo.

Apontando fragilidades no funcionamento da sustentabilidade do instituto, propunha alterações nos contratos dos funcionários e investigadores, com alargamento dos quadros permanentes (até então apenas quatro e ocupando posições diretivas) e atualização dos vencimentos. A carta termina com uma proposta de aumento do quadro do pessoal científico para o dobro (Martins, 8 jul. 1961). A petição ficaria sem resposta e evidenciava um dos grandes problemas que se apresentou no desenvolvimento do projeto do IICA: atrair corpos qualificados para a carreira de investigação científica, vista como um regime precário e pouco compensatório (Martins, 8 jul. 1961). Ao longo da sua atividade, a junta recebeu pedidos de aumento dos salários, complementos e requalificações a título individual e coletivo.

Em 1962, o diretor do IICA inicia funções docentes nos Estudos Gerais de Angola, futura Universidade de Angola. A implementação dos Estudos Gerais Universitários de Angola e de Moçambique, promulgada a 21 de agosto de 1962 (Decreto-lei n.44530, 21 ago. 1962), sucede-se após um agitado processo político entre o governo provincial de Angola e o Ministério do Ultramar em Lisboa (Sousa, 2014). Essas alterações surgem na sequência da abolição do Estatuto do Indigenato (Decreto-lei n.43893, 6 set. 1961), que supunha o acesso livre e igualitário dos angolanos à educação, à ciência e à cultura. Antes da inauguração, a 6 de outubro de 1963, em Luanda, Cannas Martins (16 maio 1963) procurou, junto da Comissão Executiva da Junta de Investigações do Ultramar, apurar quais os cursos superiores que qualificavam a admissão aos quadros científicos. Sobre esse assunto, a junta respondeu “que somente os cursos superiores mencionados no artigo 5º do decreto de Lei n. 36 507, de 17 de setembro de 1947, eram considerados cursos superiores”, o que, por conseguinte, excluía outros recém-criados. Só mais tarde, a 23 de dezembro de 1968, os Estudos Gerais adquiriram o estatuto de universidades (Decreto-lei n.48790, 23 dez. 1968), adotando as designações de Universidade de Luanda e Universidade de Lourenço Marques, respectivamente, conferindo os graus de licenciado, doutor e agregado.

Nesse contexto, surgia a proposta de alteração do regulamento do IICA enviada pela direção à junta, em Lisboa. O documento propõe uma reorganização dos quadros diretivos e a divisão em quatro departamentos: os Serviços Centrais; o Departamento de Ciências Biológicas; o Departamento de Ciências Geográfico-Geológicas; e o Departamento de Ciências Humanas. Os Serviços Centrais também integravam a gestão dos museus, o Centro de Estudos de Medicina e Cirurgia Experimental, o Centro de Documentação Científica e o Arquivo Histórico, assim como os Serviços de Administração, Seleção de Preparação e Seleção de Pessoal, o Depósito de Materiais e Oficinas, o Serviço de Fotografia e Som, e o Gabinete de Desenho.

Uma nova reestruturação aconteceu em 1970 (Decreto-lei n.463/70, 8 out. 1970), com a criação de novos departamentos com mais independência da direção (Martins, 1972). Foi introduzida a figura do subdiretor, e o instituto passou a estruturar-se em sete departamentos: (i) o Departamento dos Serviços Centrais, responsável pelos Serviços Administrativos, Centro de Documentação Cientifica, Serviços de Oficinas e Armazéns, Serviços de Fotografia e Som, Centro de Estudos de Sá da Bandeira e Gabinete de desenho; (ii) o Departamento de Ciências da Terra, com a Divisão de Geologia; (iii) o Departamento de Ciências Biológicas, com duas divisões, a Divisão de Botânica, com quatro seções (Botânica Geral, Fitogeografia, Botânica Sistemática e Farmacognósia), e a Divisão de Zoologia, com três seções (Entomologia, Ornitologia e Mamalogia), contava ainda com o Serviço de Microbiologia e o Centro de Estudos de Medicina Experimental; (iv) o Departamento de Ciências Humanas, organizado em torno da Divisão de Etnologia e Etnografia, do Gabinete de Etnossociologia, da Divisão de Geografia Humana, da Divisão de História e da Divisão de Filmoteca; (v) o Departamento de Museologia, que se ocupava da gestão do Museu de Angola, Huíla e Congo; (vi) o Centro de Cálculo Científico e Mecanográfico, com os Núcleos Científico e Técnico e o de Processamento Administrativo; (vii) o Departamento de Biblioteca. Neste último estavam integrados a Biblioteca do Museu de Angola e o Arquivo Histórico de Angola, com as seções de História, Arquivo Intermédio e Filmoteca.

Uma das principais missões do IICA era a divulgação científica, sobretudo, por meio de publicações. No entanto, o primeiro relatório indicava somente publicações na *Gazeta Agronómica*, ressalvando a preparação de um boletim para o IICA, planeado em 1957 como publicação bianual e que pretendia expor estudos realizados no instituto (Martins, 1958a).

O primeiro *Boletim do Instituto*, incluindo oito artigos, foi publicado em 1963 e organizado de acordo “com os tópicos de maior relevância económica e social, nomeadamente na agronomia e ecologia, indústria do cafeeiro, medicina e etnografia de povos do sul de Angola” (Martins, 1963, p.5). Os volumes seguintes seguiram sensivelmente a mesma linha temática, de acordo com as disciplinas das diferentes divisões de pesquisa, incluindo estudos desenvolvidos pelos próprios membros do IICA ou outros sob patrocínio da instituição.

O *Boletim bibliográfico* de 1970 elenca todas as publicações produzidas com o seu selo e outras por si vendidas, produzidas pelos organismos que absorveu ou por ele dependentes, como o Museu e Arquivo de Angola (IICA, 1971). Esses trabalhos incluíam catálogos das exposições, essencialmente por artistas plásticos portugueses, assim como outras obras de historiografia que enalteciam essencialmente o elemento colonizador (Angola..., 1945; Duparquet, 1953). Após a integração dos museus no IICA, em 1961, a edição dos catálogos cessou.

As publicações monográficas e separatas de relatórios e memórias dos trabalhos do IICA focalizaram aspetos da língua, história e cultura das populações de Angola (Estermann, 1960, 1971; IICA, 1960; Milheiros, 1960, 1967; Lima, 1964, 1972; Cruz, 1967, 1971; Barbosa, 1973; Couto, 1973). Outras, voltadas para as ciências da vida, permanecem como obras de referência sobre a paisagem e o meio ambiente (Grandvaux-Barbosa, 1970; Simões, 1971; Valente, 1973).

O instituto também divulgou a lista de todas as publicações e periódicos adquiridos pelo Centro de Documentação e Informação a partir de 1969 com uma periodicidade de quatro anos (IICA, 1969; Calado, 1974). Essa documentação lista centenas de obras monográficas, manuais, revistas, jornais e outros documentos acessíveis no instituto. O esforço no apetrechamento das unidades locais com os recursos necessários para a pesquisa não foi proporcional à divulgação de obras monográficas, que se revelou limitada por motivos financeiros. Apesar dos constrangimentos, o IICA estabeleceu uma política de permutas com diversas instituições na metrópole e em outros países, o que mantinha um fluxo atualizado sobre a ciência que se fazia fora de Angola e, ao mesmo tempo, permitia difundir os estudos realizados. O *Projeto do Programa de Trabalhos para o ano de 1965 do IICA* mostra relações de intercâmbio com 13 academias; 132 universidades, faculdades e escolas superiores; 84 associações, sociedades e comissões científicas; 209 institutos científicos; 209 centros de estudos e laboratórios; 261 museus; 69 bibliotecas e arquivos; 25 centros e serviços de documentação. Perfaziam o total de 793 instituições nacionais e internacionais (Martins, 1965, p.44). A divulgação do conhecimento produzido pelos investigadores do IICA (internos e externos) ocorria também por meio de participação em conferências subsidiadas mediante aprovação da junta.

## O fim do IICA

O IICA funcionou com o regulamento de 1970 até à dissolução da administração colonial. A partir de agosto de 1973, a correspondência emitida pela direção do IICA, em Luanda, à junta, em Lisboa, passou a ser assinada pelo diretor interino, Luís Grandvaux-Barbosa, subdiretor desde 1970. A última carta endereçada pelo IICA à junta foi enviada a 22 de outubro de 1973, sobre os requerimentos de licença ilimitada para vários trabalhadores, após uma indicação superior de que todas as decisões permaneciam adiadas à espera da reforma da junta (Informação n.2019/SA/73, 21 set. 1973). Em novembro de 1973, a junta é renomeada como Junta de Investigações Científicas do Ultramar. O decreto vinculava os institutos e centros provinciais à sua gestão e decretava a fusão dos Arquivos Nacional e Provincial ao IICA (Decreto-lei n.583/73, 6 nov. 1973). Depois de 31 de dezembro de 1973, a última entrada no índice do arquivo da junta sobre o IICA, não houve mais receção de ofícios provenientes de Luanda, nem notícia oficial das suas atividades perante a reestruturação. No final de 1973, evidências do decaimento do Estado Novo começavam a sentir-se na crescente tensão política, motivadas pela avançada e dispendiosa Guerra Colonial (Matos, Oliveira, 2023). O fim do regime anunciado pelo manifesto de António Spínola “Portugal e o futuro” a 22 de fevereiro de 1974 conduziria a um golpe militar frustrado nas Caldas da Rainha, a 16 de março (Soares, 16 mar. 1974). A revolução de 25 de abril de 1974 abriu o caminho à progressiva desativação dos organismos associados ao Ministério do Ultramar, como era o caso dos governos-gerais e da junta, e, por conseguinte, do IICA e dos seus subsidiários.

Em julho de 1974, a Assembleia Magna dos Funcionários do IICA reuniu os investigadores para emitir um parecer coletivo sobre o futuro daquele organismo ministerial, dando resposta a um pedido dirigido pela junta. O parecer coletivo assinado por Marcelo de Sousa Vasconcelos e assistido por Lapido Loureiro traça não só uma história do instituto, mas também um plano de futuro, que obedecia, *grosso modo*, às mesmas linhas temáticas do IICA, afirmando que “a ocupação científica do país é por ora ainda irrisória, e deverá desenvolver-se em proximidade com as atividades económicas do país” com novas perspetivas “de e para Angola” (Vasconcelos, 1974, p.11). Neste manifesto, o corpo de investigadores defendia a transição dos quadros e património, mas os recursos humanos que compunham o IICA eram, essencialmente, provenientes de Portugal. Após a independência, a 11 de novembro de 1975, muitos dos investigadores do IICA integrariam um movimento migratório de grande impacto em Portugal; eles foram chamados de “retornados” (Duarte, 2019). Os próprios Vasconcelos e Lapido Loureiro, que dirigiram o manifesto, regressaram a Portugal mantendo vínculo com o funcionalismo público português.

A extinção do Ministério do Ultramar conduziu à remodelação das instituições portuguesas. O esforço governamental se concentrou na acomodação desses recursos humanos entre os corpos científicos que atuavam na junta, em Lisboa. A combinação dos corpos científicos da Junta e dos organismos provinciais justificou a manutenção e o estudo dos arquivos levados durante as missões coloniais. Em 1976, a junta passou para a tutela do Ministério da Cultura e da Ciência, mas foi extinta a 31 de dezembro de 1979, dando lugar ao Laboratório Nacional de Investigação Tropical. O diploma estipulava a transição direta da orgânica, do património e de trabalhadores, assim como direitos e responsabilidades da junta (Decreto-lei n.532/79, 31 dez. 1979). Esse decreto omitia o Museu de Etnologia e património associado, o que viria a ser retificado em 1982 (Decreto-lei n.105/82, 8 abr. 1982), assim como a renomeação para Instituto de Investigação Científica Tropical, incluindo a criação de um museu, que nunca veio a ser concretizada. Em 1988, a tutela do instituto foi transferida para o Ministério do Ordenamento do Território e Gestão, acabando por resultar na passagem do Museu de Etnologia para o recém-criado Ministério dos Negócios Estrangeiros. Esse processo de desmembramento das coleções e centros de investigação a partir de 2003 (Decreto-lei n.297/2003, 21 nov. 2003) finalizou-se em 2015, com a fusão do património e recursos humanos à Universidade de Lisboa (Matos, 2021). Herdando as coleções, os institutos e as pessoas, a continuidade da investigação foi legitimada no âmbito das políticas públicas de desenvolvimento e cooperação internacional com os países de língua oficial portuguesa. O Estado português procurou acomodar os “cientistas retornados”, com a transplantação das suas atribuições para Lisboa. Evidências desse processo encontram-se na própria estrutura do Instituto de Investigação Científica Tropical, com divisões decalcadas dos centros existentes nos institutos de Angola e Moçambique (Lobato, 1983). Em muitas áreas, a investigação continuou graças à colaboração entre colegas em Angola e Portugal (Crawford-Cabral, Veríssimo, 2005; Crawford-Cabral, 2009).

Na transição governamental, o IICA foi gradualmente integrado no novo sistema científico de Angola, inicialmente como Centro Tecnológico Nacional mais tarde renomeado Centro Nacional de Investigação Científica (CNIC), sob a tutela do Ministério da Educação e Cultura. Nessa fase, foi dirigido pelo antigo diretor do centro do IICA no Lubango, Óscar Azancot de Menezes (1965), que assumiria a transição dos organismos da ciência colonial na independência.

Em 1999, a tutela do CNIC e todo o património do ex-IICA foram transferidos para o Ministério da Ciência e Tecnologia, consistindo numa reformulação ministerial que separou definitivamente as estruturas antes albergadas no IICA (Decreto Executivo Conjunto n.97/99, 3 set. 1999). Organismos subsidiários como os centros agronómicos e os arquivos passaram para a tutela do Ministério do Ambiente e do Ministério da Administração Pública, respetivamente. O centro regional do Lubango foi extinto e deu lugar ao Arquivo e Biblioteca Provincial. As coleções biológicas foram salvaguardadas pelo ISCED, localizado no edifício da antiga Universidade de Angola. Atualmente, as coleções são exibidas no Centro de Ciência da Huíla (2020). O Museu da Huíla transformou-se em Museu Regional da Huíla (1975), mantendo a sua coleção intata. Inicialmente dependente da Direção Provincial da Cultura da Huíla, passou para a tutela do Ministério da Cultura de Angola (Decreto Executivo n.5/14, 8 jan. 2014).

Em Luanda, o decreto executivo n.15/99, de 8 de outubro de 1999, aprovou o estatuto orgânico do Ministério da Ciência e Tecnologia, dando conta, no artigo n.2, da efetivação da transferência da tutela do CNIC e de todo o património do extinto IICA para aquele ministério. Contudo, só em 2003 é oficialmente criado e aprovado o seu estatuto orgânico (Decreto Executivo n.91/03, 7 out. 2003), confirmado por decreto presidencial em 2011 (Decreto Presidencial n.251/11, 26 set. 2011). O diploma define uma instituição pública dedicada à promoção e à realização de investigação científica multidisciplinar em Angola. No campo da museologia e património cultural, novos museus foram criados a partir de depósitos e coleções do IICA de Luanda, com a reconversão do Museu de Angola em Museu Nacional de Antropologia e a criação do Museu Benguela, e a fundação do Museu Nacional de Arqueologia.

Em Portugal, as coleções dos diferentes centros de investigação (Geologia, Agronomia, Pré-história e Arqueologia, Botânica) do antigo Instituto de Investigação Científica Tropical encontram-se distribuídas pela Universidade de Lisboa. Os arquivos e o património imóvel, incluindo vários palácios e jardins botânicos em Belém e na Ajuda, são atualmente geridos pela Reitoria/Serviços Centrais (Despacho n.7680/2016, 9 jun. 2016), na esfera de atuação do Museu de História Natural e da Ciência (Despacho n.4283/2017, 18 maio 2017). Novas colaborações para a conservação e estudos dos patrimónios partilhados encontram-se em curso entre o Ministério da Cultura de Angola e a Universidade de Lisboa.

## Considerações finais

Neste artigo procurou-se traçar a história do IICA e responder às questões inicialmente lançadas sobre a criação, missão e funcionamento do instituto dentro da história das instituições coloniais portuguesas, observando igualmente aspetos do seu declínio e transformação. De acordo com as fontes documentais, o IICA foi fundado em conjunto com o Instituto de Investigação Científica de Moçambique e os Institutos de Medicina Tropical em 1955, consolidando o plano de ocupação científica do ultramar que vinha sendo debatido por académicos e políticos em Portugal. Esse plano começou a desenhar-se no seio da política colonial do Estado Novo, com a promoção das missões de reconhecimento geográfico e geológico. A origem do IICA remonta ao projeto de definição e conformação do conceito de Ultramar introduzido pelo Estado Novo no período pós-Segunda Guerra Mundial, como mecanismo de manutenção de um poder em declínio face aos movimentos de independência das nações africanas.

Como extensão do território português, entendia-se que os territórios ultramarinos mereciam o mesmo tipo de estruturas e atividades, o que exigia a descentralização da administração colonial e a criação de organismos dedicados às várias áreas científicas, complementando as necessidades económicas de Portugal na ocupação do território africano. O IICA constituía um instrumento de ocupação efetiva, que se pretendia atrativo a quadros superiores, que iriam desenvolver ciência do mais alto nível. Na realidade, a correspondência entre a direção do IICA e a Comissão Executiva da Junta, que, embora não facultasse apoio financeiro direto (que era assumido pelo Governo Central de Angola), emitia parecer a todas as atividades, mostra que as oportunidades de progresso dentro do IICA eram limitadas, assim como os recursos para a sua ação. O IICA funcionou sobretudo com quadros importados, cuja maioria regressou a Portugal depois da independência de Angola.

Durante a sua atividade, a organização e gestão do IICA centraram-se, essencialmente, na figura do seu diretor Virgílio Cannas Martins e dos chefes de divisão por ele nomeados. Toda a atividade do IICA era reportada via postal à junta, em Lisboa. A atividade científica, que se pretendia livre, era inevitavelmente controlada pela comissão executiva na metrópole e pelos diretores dos centros metropolitanos, sempre enquadrada nas estratégias ideológicas de membros políticos, intelectuais e cientistas externos ao território angolano. Durante a sua atividade, o corpo de funcionários e investigadores debateu-se muitas vezes com questões relacionadas aos direitos do trabalho, progressão na carreira e aumento dos salários.

Apesar disso, o IICA desempenhou um papel de destaque no percurso da ciência em Angola até 1975. Nos períodos decorridos, as reformas dirigidas pela governação da República de Angola transformaram os despojos do instituto no atual CNIC. Contudo, vasta documentação sobre os departamentos do IICA e as suas atividades permanece em localização incerta. No legado bibliográfico do IICA avultam as mais variadas produções científicas patrocinadas pelo instituto na forma de monografias e artigos. Em alguns domínios como a agronomia, a etnografia e a sociologia constituem obras únicas. Após 1975, o património do IICA, já de alguma forma espartilhado entre Luanda e Lubango, nas estações de intervenção agronómica e sociológica, persistiu mediante a preservação e conservação das coleções de referência das várias divisões, incorporadas no sistema de ensino superior e museus da República Popular de Angola.

## Data Availability

Não estão em repositório.
